# The Relation Between GABA and L-Arginine Levels With Some Stroke Risk Factors in Acute Ischemic Stroke Patients

**Published:** 2016-06-28

**Authors:** Mohsen Hosinian, Durdi Qujeq, Alijan Ahmadi Ahangar

**Affiliations:** 1 *Department of Clinical Biochemistry, Babol University of Medical Sciences. Babol, Iran.*; 2 *Student Research Committee, Babol University of Medical Sciences, Babol, Iran.*; 3 *Cellular and Molecular Biology Research Center (CMBRC), Health Reasearch Institute, Babol University of Medical Sciences, Babol, Iran.*; 4 *Department of Neurology, Ayatollah Rouhani Hospital, Babol University of Medical Sciences, Babol, Iran.*; 5 *Mobility Impairment Research Center, Babol University of Medical Sciences, Babol, Iran.*

**Keywords:** Gamma aminobutyric acid (GABA), arginine, acute ischemic stroke

## Abstract

Changes in extra and intracellular neurotransmitter amino acids concentration in the early stage of acute cerebral ischemia have been reported. In this the study, serum level of gamma aminobutyric acid (GABA) and L-Arginine in acute ischemic stroke patients was assessed. 60 patients with acute ischemic stroke and sixthy healthy volunteers as a control group were assessed. Serum GABA was measured with modified enzymatic method and serum L- Arginine was measured by modified Sakaguchi method. Serum GABA level in stroke cases was lower than that of the control group. There was no relationship between GABA level and age or gender. Also, no significant correlation was observed between GABA levels with ischemic stroke risk factors such as smoking, diabetes mellitus, and hypertension. Serum L- Arginine level in patients was slightly increased in comparison with control group. There was a positive relationship between serum L- Arginine level and acute ischemic stroke risk factors. Serum GABA level was reduced in patients and had no correlation with acute ischemic stroke risk factors.

Previous studies demonstrated that in mammalian central nervous system, gamma aminobutyric acid (GABA) is a major inhibitory neurotransmitter (1). In the brain, GABA binds to receptors that cause hyperpolarization in neurons. There are two classes of GABA receptors: GABA a receptor, is a part of ligand-gated ion channel complex and GABA b receptors, metabotropic receptors which are G protein coupled receptors (2). It has been reported that GABA's major role is to reduce neural excitability in human. It also regulates muscle tone (3). The implication of excitotoxicity and the glutamate-calcium cascade in the development of neural injury after brain ischemia were proven previously(4-7). Some experimental evidence suggests that after ischemic stroke, glutamate efflux increases in brain and causes intracellular Ca2+ increase, resulting in neuronal injury. Ischemic stroke also triggers cytotoxic cascades leading to expression of cell death (7). GABA as an inhibitory neurotransmitter can counteract glutamate effect and reduces neural injury. Correspondingly, there is increasing interest in studies of excitatory and inhibitory neurotran-smitter levels in body fluid of acute ischemic stroke patients (8). Some studies assessed the level of excitatory and inhibitory neurotransmitters in cerebrospinal fluid (9, 10), extracellular fluid (11) and plasma (12-14).

L-Arginine is an amino-acid that has an important role in cell division, immune function and release of hormones. It is synthesized from citrullin by two cytosolic enzymes argininosuc-cinate synthetize (ASS) and argininosuccinate lyase (ASL). Some data have revealed that L-Arginine is a precursor of nitric oxide (NO). NO has both neurotoxic and neuroprotective roles. In this regard, NO generation has been related to brain damage in acute ischemic stroke (15). Researchers reported that GABA receptor agonists had neuroprotectant effect in reducing infarct size (12) and after stroke, the patients had lower GABA levels. These results suggest that the function of GABA is decreased outside the infarct. Therefore, further researches are needed for elucidation of the possible role of GABA changes in stroke. In this study, we evaluated serum GABA and L-arginine levels in acute stroke patients and their relationship with acute ischemic stroke risk factors.

## Materials and methods

**Subjects**

A total of 60 consecutive acute ischemic stroke patients who were admitted in emergency department of Ayatollah Rouhani Hospital, Babol, and 60 healthy volunteers were included in this study. The number of samples was based on the results of other papers, average measured factors and the prevalence of acute ischemic stroke. The diagnosis of acute ischemic stroke was based on characteristic clinical manifestations together with physical and neurological examination, which was confirmed by a neurologist. Inclusion criteria were: acute ischemic stroke, first episode of stroke, admission within 24 hours after the onset of symptoms, and persistence of neurological deficit on admission. Patients with renal or liver diseases, gout and metabolic disorders such as hypoglycemia with hemiparesy or other focal neurological findings, as well as hemiparetic patients due to trauma, or subject taking supplements in early past, were excluded.

All subjects gave informed consent and the study protocol was approved by Babol University Ethics Committee. Clinical characteristics including age, gender, and history of diseases such as hypertension, diabetes mellitus, hyperlipidemia and past history of smoking were recorded based on medical records of the patients. Blood sample was taken from all patients with acute ischemic stroke during 24 hours of admission and serum was separated and immediately stored at −80 °C.

**GABA assay**

Serum GABA was measured by modified enzymatic method previously described (16). Briefly, 2.6 ml pyrophosphate buffer (0.1 mM, pH= 8.6), 0.15 mM NAPD+NADP^+^ (4 µM, pH= 8), 0.15 ml of GABAse solution with 0.25 IU and 100 µl of GABA standard solution or deproteinized serum were mixed and absorption of solution was read at 540 nm as initial absorption (A0). Then, 0.15 ml alpha-ketoglutarate (0.2 M, pH= 7.4) was added and absorption of solutions was measured as a second absorption (A1). The difference of (A0) and (A1) was read and GABA concentration was calculated by using standard curve.

**L-arginine assay**

Serum L-Arginine was measured by modified

Sakaguchi method which was previously published (17). Briefly, 100 µl deprotenized serum or L-Arginine standard solution was mixed with 100 µl 0.15 mM sulfosalicylic acid –oxin solution. Then, 5 µl NaOH (2.5%) was added and the solution was refrigerated in ice water for 15 min. Then, 0.5 ml of hypobromite 0.25 mM was added and absorption of solution was measured at 520 nm. The amount of L-arginine was calculated by using standard curve.

**Statistical analyses**

Results for the subject group followed a normal distribution, we used parametric methods, two independent test, and Mann-Whitney U test to compare the groups. Also, Spearman correlation test was used to evaluate the correlation values. Results are expressed as mean±SD, and p<0.05 was considered as statistically significant and subject groups were compared using one–way analysis of variance.

## Results

In the present study the mean age of the patients was 64 years (SD=7.2) and 23 of the subjects were females. Clinical characteristics of patients and control group was shown in Table 1. Serum GABA level in patients was lower than control group as shown in Table 2. There was no relationship between serum GABA and diabetes mellitus (rs=-0.014, P= 0.879), hypertension (rs=-0.087, P=0.346), smoking (rs=-0.098, P=0.288). Serum L-Arginine level in patients slightly increased compared to control group as shown in Table 2. There was a positive relationship between serum L-Arginine and diabetes mellitus (rs=0.448, P=0.000), smoking (rs=0.523, P=0.030), hyperten-sion (rs= 0.378, P= 0.044).

## Discussion

One important finding in the current study is that, as compared with normal subjects, serum GABA level decreased in acute ischemic stroke patient group. Furthermore, it is clear that due to decrease of GABA level, various anxiety disorders are triggered, because GABA plays an important role in overall brain function. In this regard, researchers reported that the use of GABA receptor agonist for the treatment of patients with acute ischemic or hemorrhagic stroke is not suitable (12). In another study, researchers reported that pre- ischemic treadmill training decreased glutamate release and increased GABA release during the acute phase of cerebral ischemia/reperfusion (13). It is well known that GABA plays an important role in modulating brain repair (14). After ischemia, a reduction in GABA-ergic neuronal activity, an increase in neuronal or glial GABA uptake was reported (18, 19). In addition, the regulation of the activity of dopaminergic system is under the influence of GABA ergic and glutaminergic system (20)**.**

**Table 1:** Clinical characteristics of patient and control groups.

Parameter	Patients (n=60) Mean (SD)	Controls (n=60) Mean (SD)	P value
Age	64 (7.2)	62 (6.7)	
Female/ male	23/37	25/35	
Systolic BP (mmHg)	142 (17.2)	128 (16.2)	<0.001
Diastolic BP (mmHg)	88.4 (20.2)	79 (15.3)	<0.001
Total cholesterol (mg/dl)	197.45 (40.45)	195.36 (47.50)	>0.05
Triglycerides (mg/dl)	178.5 (40.0)	138.68 (43.3)	<0.001
HDL cholesterol (mg/dl)	53.25 (20.23)	59.56 (22.62)	<0.001
Hypertension (%)	37 (61)	17 (28)	<0.001
Diabetes (%)	29 (48)	20 (33)	<0.01
Smokers (%)	22 (36)	16 (26)	<0.001

BP: blood pressure; HDL: high density lipoprotein

**Table 2:** Serum GABA and L-Arginine levels in patient and control groups.

Parameter	Patients	Controls	P-value
GABA (µg/ml)	266 (35)	304 (42)	0.739
L-arginine (mg/ml)	188 (21)	186 (37)	0.328

Data are presented as Mean (SD)

We hypothesized that the reduction of serum GABA level following acute ischemic stroke can be attributed to the change of GABA metabolism. Two enzymes play an important role in GABA production. GABA is produced by glutamate decarboxylase through the decarboxylation of L-glutamate or catalysis from L-glutamine by glutaminase enzymes.

GABA might act as a neuroprotector for acute ischemic stroke, although its action mechanism remains unclear. In the present study, we observed that serum L-Arginine slightly increased in acute ischemic stroke patients. Also, it has a positive correlation with acute ischemic stroke risk factors. This observation is in disagreement with the results obtained by other investigators (21) Armengou et al. assessed plasma and cerebrospinal fluid (CSF) L-Arginine in 268 acute stroke patients within 8.2 hours after stroke onset. They found that plasma and CSF L-Arginine levels decreased in patient group. They observed positive relationship between plasma and CSF L-Arginine levels (22). in addition, Rashid et al. evaluated nitrate and nitrite, L-citrulline, cGMP and L-Arginine in acute stroke patients. They found that L-Arginine level reduced independently from patients' feeding status (23). We suggest that the slight increase of L-Arginine levels in acute stroke may be due to biochemical reactions that cause the increase of extracellular L-Arginine concentrations. The slight increase of L-Arginine may also be related to diffusion of arginine through the blood–brain barrier in acute ischemic stroke patients. Armengou et al. found a negative correlation between nitric oxide metabolites (NOx) and L-Arginine concentrations in CSF within 24 hours of stroke onset (22). Another cause of serum L-Arginine increase may be its own metabolism in the acute phase of stroke. Another possibility of change in L-Arginine levels in blood may be related to the acute-phase reaction or systemic causes like concomitant infections (24). Researchers reported that metabolites of the L-Arginine pathway were elevated in the very acute phase of ischemic stroke (25). Collectively, some investigators have observed an association of serum markers levels with stroke (26-28). Our study corroborates these results. Consequently, it is important to investigate the putative mechanisms involved in serum marker changes.

The present study had some limitations such as being a single center study. Also, the number of studied subjects was relatively low.

In conclusion, serum GABA levels were reduced in acute ischemic stroke patients and had no correlation with acute ischemic stroke risk factors. However, serum L-Arginine levels were slightly increased in acute ischemic stroke patients. In addition, it has a positive correlation with acute ischemic stroke risk factors.
